# Older patients’ perspectives on illness and healthcare during the early phase of the COVID-19 pandemic

**DOI:** 10.1177/09697330211072362

**Published:** 2022-03-03

**Authors:** Nina Jøranson, Anne Kari Tolo Heggestad, Hilde Lausund, Grete Breievne, Vigdis Bruun-Olsen, Kristi Elisabeth Heiberg, Marius Myrstad, Anette Hylen Ranhoff

**Affiliations:** Faculty of Health Studies 87368 VID Specialized University Oslo, Norway; Faculty of Health Studies VID Specialized and Senior researcher, University of Oslo, Center for Medical Ethics, Oslo, Norway; Faculty of Health Studies VID Specialized University Oslo, Norway; Faculty of Health and Social Sciences, Institute of Nursing and Health Sciences, 177041 University of South-Eastern Norway, Drammen, Norway; Department of Medical Research, Bærum Hospital, 155273 Vestre Viken Hospital Trust, Drammen, Norway; Department of Physiotherapy, 60499 OsloMet - Oslo Metropolitan University, Oslo, Norway; Department of Medical Research, Bærum Hospital, 155273 Vestre Viken Hospital Trust, Drammen, Norway; Department of Internal Medicine, Bærum Hospital, 155273 Vestre Viken Hospital Trust, Drammen, Norway; 11316 Diakonhjemmet Hospital Trust, Department of Medicine, Oslo, Norway; 1658 University of Bergen, Clinical institute 2, Bergen, Norway; 25563 Norwegian Institute of Public Health, Oslo, Norway

**Keywords:** Older people, ethics, COVID-19, vulnerability, access to healthcare, priorities

## Abstract

**Background:**

Equal access to healthcare is a core principle in Norway’s public healthcare system. The COVID-19 pandemic challenged healthcare systems in the early phase – in particular, related to testing and hospital capacity. There is little knowledge on how older people experienced being infected with an unfamiliar and severe disease, and how they experienced the need for healthcare early in the pandemic

**Aim:**

To explore the experiences of older people infected by COVID-19 and their need for testing and hospitalisation.

**Research design:**

An explorative and descriptive approach, with qualitative interviews conducted in October 2020.

**Participants and research context:**

Seventeen participants above 60 years of age hospitalised due to COVID-19 during spring 2020 were recruited 6 months after discharge.

**Ethical considerations:**

Ethical approval was granted by the Regional Committee for Medical and Health Research Ethics in South-Eastern Norway (155425).

**Findings:**

The main finding was that the informants experienced vulnerability and arbitrariness. This finding was supported by three sub-themes: experiences with a severe and unfamiliar disease, the strict criteria and the importance of someone advocating needs.

**Discussion:**

Participants described varying access to healthcare. Those who did not meet the national criteria to be tested or hospitalised struggled against the system. Findings reveal arbitrary access to healthcare, in contrast to Norway’s ethical principle of fair and just access to health services. Moreover, to access and receive necessary healthcare, informants were dependent on their next-of-kin’s advocacy.

**Conclusion:**

Even when dealing with an unfamiliar disease, health professionals’ assessments of symptoms must be performed with an ethical obligation to applicate competent appraisal and the exercise of discernment; this is in line with care ethics and ethical standards for nurses. These perspectives are a significant part of caring and the intension of doing good.

## Introduction

The early phase of the COVID-19 pandemic challenged healthcare systems around the world.^1^ Older people have been especially vulnerable to infection with SARS-CoV-2, and many of the oldest have faced serious illness because of this strain.^2-4^

Despite relatively few SARS-CoV-2 cases in the early phase of the pandemic, Norway implemented national lock-down measures in the middle of March 2020 in order to mitigate the pandemic.^5^ Estimates indicated a potential peak of infections in April/May 2020 that threatened the expected capacity of healthcare services, especially in acute care.^6^ However, compared to many other countries, relatively few patients have been hospitalised due to COVID-19 in Norway.^6^ In contrast to many other countries, Norway has handled the crisis well, due to strong national institutions, a good economy and a high-trust society with a reliable and professional bureaucracy.^7^ This is also in line with how the Norwegian healthcare system has been described – indeed, a newly published report states that Norway has one of the best healthcare systems worldwide.^8^

Norway has a publicly financed healthcare system, in which specialized healthcare services are provided almost free of charge.^7^ Here, one of the core principles is that all citizens should have equal access to appropriate healthcare when needed.^9^ In addition, Norwegian law states that all health care should be safe and appropriate based on patients’ needs. To ensure that the principle of fair and just healthcare allocation is upheld, the Norwegian government has a long tradition of developing and implementing criteria for prioritisation. The criteria are *severity of condition*, *benefit of intervention* and *resources*, and should be complied with by authorities and professionals when allocating healthcare, on micro, meso and macro levels.^10^ Nurses’ responsibility regarding the criteria is outlined in the International Council of Nurses’ (ICN), ethical standards: for example, point 1.7 – ‘nurses advocate for equity and social justice in resource allocation, access to healthcare and other social and economic services’.^11^ Further, one point derived from Norwegian Nursing Association’s ethical standards is in particular point 6.3 – ‘the nurse contributes to ensuring prioritisations that benefit the patients in greatest need of nursing care’; and point 6.2 – ‘the nurse contributes actively to meet the special needs of vulnerable groups concerning health and care services’.^12^ The professional ethical standards underline the care responsibility of each nurse in order to advocate for patients who are not given prioritisation based on professional considerations and discerning judgements.

In the early phase of the pandemic, health policy authorities had limited knowledge about the disease and its potential development. Consequently, the Norwegian government developed COVID-19 guidelines for the prioritisation of patients and access to healthcare.^13^ Criteria for testing until the middle of March 2020 included anyone with an acute respiratory infection and symptoms (coughing, shortness of breath and fever), and who had (or were in close contact with someone who had) recently visited an area with a documented spread of COVID-19 (e.g. China, Northern Italy and Austria).^5^ From the middle of March, people with COVID-19 symptoms in need of hospitalisation were given testing priority.^6^ Hospital admission rates in Norway declined in the early phase of the pandemic. Many patients with suspected or diagnosed COVID-19 were treated out-of-hospital, including many older and frail patients.^14^

## Previous research

International studies mainly report findings concerning the experiences of patients with COVID-19 during hospitalisation. Studies from China in the early phase of the pandemic describe patient experiences of fear, stigma and uncertainty when hospitalised with COVID-19.^15, 16^ Moreover, a qualitative study from Denmark found that older patients experienced anxiety and a loss of dignity and autonomy during hospitalisation.^17^

Research also shows that access to and delivery of healthcare were challenged during the pandemic, especially related to the access to ventilators^18^ Both high income and low-income countries experienced barriers to COVID-19 testing.^19, 20^ In Norway, health trusts were forced to change routines daily in response to the unpredictable situation and to mitigate the expected rapid increase in numbers of infections and COVID-19 patients.^5, 6^ Consequently, health professionals experienced several ethical dilemmas around establishing prioritisation during the pandemic, especially in the first phase.^21, 22, 23^ It was decided that patients suffering from COVID-19 would be prioritised over all other patient groups, due to a fear of overwhelming the healthcare services.^14^

Most COVID-19 research in this area has primarily focused on health professionals’ experiences with the healthcare system, with patient experiences receiving limited attention. There is little knowledge on how older patients in Western countries experienced access to healthcare services and hospitalisation during this time, especially in the early phase of the pandemic. In addition, the important role of next-of-kin in ensuring access to healthcare services on behalf of the very ill is underexplored.

This paper reports results from a sub-study of a larger cohort study investigating change in health-related quality of life, functional decline and long-term mortality in older people 6 months after hospitalisation due to COVID-19 in the early phase of the pandemic. The cohort study describes a decline in quality of life in more than half of the participants, while more than 30% show impaired ability in activities of daily life, reduced mobility and increased pain/discomfort.^4^ The current sub-study, however, investigates participant experiences with having symptoms of COVID-19 at home before being admitted to hospital.

## Aim

The aim of the current study was to explore older patients’ experiences with having COVID-19 at home and their access to testing and hospitalisation in the first phase of the pandemic.

## Design and methods

The study had an explorative and descriptive design and was part of a multi-centre cohort study in south-eastern Norway.

## Participants and context

Patients aged 60 years and older who had been admitted to 1 of 4 general hospitals in south-eastern Norway during the first phase of the pandemic (1 March to 1 July 2020) were invited to participate in the main study. See Walle-Hansen et al.^4^ for further details. Two of the four hospitals are university hospitals and two are not: Patients from the latter who were invited to a follow-up consultation around 6 months after hospitalisation were invited to participate in the sub-study. Those who were considered physically and cognitively able to participate in an interview were invited by the project geriatrician to participate in the sub-study after completing the follow-up consultation. A total of 17 accepted the invitation and formed a convenience sample, equally distributed between the two hospitals (see Table 1 for an overview of participants). Both hospitals are situated in the south-eastern part of Norway.

**Table 1:** Characteristics of participants.

Informant	Age (range)	Sex (male/female)	Education (years)	Cohabitant
1	70–74	m	>12 y	yes
2	75–79	m	>12 y	yes
3	95–99	m	>12 y	no
4	60–64	m	>12 y	yes
5	75–79	f	>12 y	no
6	60–64	f	>12 y	yes
7	70–74	m	>12 y	no
8	60–64	m	>12 y	yes
9	60–65	f	>12 y	yes
10	70–75	f	>12 y	Yes
11	65–70	m	>12 y	yes
12	65–70	m	>12 y	yes
13	85–90	f	<12 y	yes
14	90–94	m	>12 y	yes
15	80–84	m	>12 y	yes
16	80–84	f	<12 y	yes
17	60–65	m	>12 y	yes

## Interviews

The research was conducted with a qualitative approach using semi-structured individual interviews. We conducted all interviews between September and November 2020, and the participants were given the choice of being interviewed at home or in the researchers’ office. Each interview lasted for one to one-and-a-half hours, and pairs of researchers (VBO/KEH, AKTH/NJ, HL/GB) were present each time. A total of 14 interviews were conducted, 11 consisting of individual interviews and three consisting of couples, where both spouses had been hospitalised with COVID-19 during the same time period. Interviews were based on a thematic interview guide with open-ended questions. The interviews were audio-recorded and transcribed verbatim by a professional transcriptionist.

## Data analysis

We analysed the data using thematic analyses. Thematic analysis is a cluster of different thematic approaches used to identify, interpretate and report various aspects of research topics discovered in the data.^24-26^ Our thematic analysis process was inspired by the following six steps proposed by Braun and Clarke^25^: (1) becoming familiar with the material by reading it openly and writing ‘familiarization notes’; (2) systematic data coding; (3) generating initial themes from coded and collected data; (4) developing and reviewing themes; (5) defining, refining and naming themes; and (6) writing the report. Thematic analysis developed as a procedure is however a simplification of the analysing and reporting process itself. Analysing qualitative data in this way is an interpretative and reflexive process, in which understandings are developed by moving back and forth between the six steps, facilitating engagement with and deep reflection around the data.^22^ The six phases are to a certain level blend together which made the analytic process becoming what Braun and Clarke describe as a reflexive thematic analysis.^24-26^

The researchers wrote reflections on their experiences of the interviews as fieldnotes. While the interview transcripts formed the basis of our analysis, the fieldnotes were an analytic resource contributing to our reflexive engagement in the thematic analysis. The interview transcripts were read thoroughly by the authors and preliminary themes were generated from initial codes. In particular, the development from preliminary themes to final themes was an ongoing process of developing, reviewing, defining, refining and naming. This reflexive process also continued during the reporting phase, as the preliminary (fragmented and overlapping) themes and sub-themes were developed into themes that were substantially consistent.

## Ethical considerations

The larger cohort study was planned by geriatricians in several EU countries and Norway during the beginning of the pandemic, to investigate outcomes in older adults with COVID-19. The design was an observational study with no experimental interventions,^27^ and was approved by the Norwegian Geriatric Society. Ethical approval was granted by the Regional Research Committee in Eastern Norway (no. 155,425) and complies with the Declaration of Helsinki.

This sub-study included only individuals who were able to participate in the interviews and could provide informed consent. There were no risks for the participants, except for the burden of participating in an interview. Participants were assured confidentiality and informed that they could withdraw from the study at any time, without consequences. All participants received written information about the study and gave written informed consent.

## Results

Below, we organised the results under one main theme, *vulnerability and arbitrariness*, which comprises three sub-themes, *experiences with a severe and unfamiliar disease*, *the strict criteria,* and *the importance of someone advocating needs*.

### Vulnerability and arbitrariness

Our findings show how vulnerable patients may be when encountering an unfamiliar and uncontrollable pandemic. Patients were totally dependent on the healthcare system and the ways in which health professionals responded to their symptoms and needs, and how healthcare was allocated. Several informants described feelings of vulnerability in their experiences of life-threatening situations. Some reported experiencing that their situation was not understood well enough by health professionals, despite the clear severity of their symptoms. Several also described how providers’ professional judgement appeared to be overridden by their interpretation of very strict allocation criteria, with regards to testing and/or hospitalisation. However, others explained that they received help quite easily. Access to healthcare therefore seemed to be arbitrary among our informants. Moreover, for those who experienced symptoms but who had difficulty accessing healthcare, their close relatives were extremely important – and indeed may have saved their life.

### Experiences with a severe and unfamiliar disease

COVID-19 was a new and very serious disease, and although most of the common symptoms were known, they were difficult to interpret, and difficult to distinguish from a regular flu. Many of our informants described experiences of severe illness, and of an unfamiliar disease with symptoms they did not recognise from earlier diseases.

A striking finding was the variety of COVID-19 symptoms experienced by the older informants in the initial phase of the pandemic. Few reported symptoms of flu – such as fever, coughing and difficulty breathing – but rather other symptoms. One explained that she had a severe headache with a continuous, concerning throbbing, making her suspect that there was something seriously wrong with her brain. In addition, she developed urinary incontinence and had blood in her faeces: symptoms she had never before experienced. Another informant reported tiredness and a feeling of heaviness in his body; he initially suspected that his regular hay fever was responsible. These kinds of symptoms, which did not quite correspond with the known symptoms of COVID-19, made it difficult for older informants to understand that they had been infected. Several informants also found it challenging to find the right words to describe their situation:

*I knew that I was seriously ill. That was my experience. I have never felt like this. I woke up, and felt my body…was it a flu? But it wasn’t. It was something else. (Informant 9)*

As another informant stated succinctly: *‘It was weird—really, really weird’* (Informant 8). This lack of understanding resulted in confusion, which made the participants hesitant to request healthcare. They chose to stay at home, and their exacerbating symptoms affected both their physical capacity and mental state. A strong feeling of feebleness was reported by all, and it became harder to breathe. Some of them described being close to giving up:

*I told myself, ‘Now you are very sick, and you have given up’. Even not when I fought cancer did I give up this way. I just thought, ‘Now I am terribly ill’. Therefore, it was a relief to be hospitalized, for someone to keep me under surveillance. I didn’t realize myself that it really was going that bad with me*. (Informant 6)

Our informants also described struggling to eat and drink, which was therefore often neglected. As their symptoms continued to exacerbate, a feeling of indifference to the whole situation became prominent.

### The strict criteria

Another striking finding experienced by several informants concerned the lack of preparedness among the Norwegian healthcare system early in the pandemic. In the informants’ experience, it was not only the hospitals that were unprepared: the healthcare system, as a whole, was insufficiently prepared to meet the great public demand.

*Before the pandemic hit us, there were several times on national television where the Norwegian Institute of Public Health stated that they were well-prepared. ‘Everything is in order’. Right? That was what they said. But nothing was actually prepared that really worked*. (Informant 10)

Our informants experienced a lack of equipment, both for infection control and for testing. Due to the lack of testing equipment in the early phase of the pandemic, the healthcare system had insufficient capacity to test all those displaying symptoms. One informant (who was infected very early) experienced multiple denials, from several places, when he requested a test; moreover, no doctor would perform home visits due to fear of contagion. Many of our informants experienced what they described as a chaotic situation, as illustrated by Informant 9, ‘*So I was a frontline soldier, I call myself. I was in the front row*’.

Several informants talked about how, despite having an obvious illness and severe symptoms, they had to fight against the system to get a test that would prove that they were suffering from COVID-19. Without a test, they were not considered for admission to hospital, even though they needed professional care. The participants described the health professionals as interpreting the testing criteria in a very strict way, resulting in few opportunities for professional consideration and individual judgement. If the informants did not meet any of the national testing criteria, they were usually denied a test, despite displaying severe symptoms (such as a very high fever, e.g. 40–41°C, even for several weeks). Some informants believed that they would have died had they not pushed back. As one informant stated, ‘*Only ski tourists and health professionals were tested. And the rest of us, we just had to do our best*’ (Informant 10).

Moreover, even once they were tested and found to have COVID-19, this did not guarantee that they would be hospitalised, despite the severity of their symptoms. As Informant 1 stated, ‘Even when I had a positive test result, they sent me home’. Some informants described how they struggled for weeks, alone or with their next-of-kin, before being admitted to hospital – even after testing positive and displaying severe symptoms. One older woman explained that she overheard the health professionals in the ambulance discussing whether she really was ill enough to be hospitalised. She did not feel safe and could not relax until she received a bed inside the hospital.

### The importance of someone advocating needs

Several informants talked about how, though they were very unwell, they were unable to assess their needs nor take the initiative to contact the healthcare system. One informant, whose husband was hospitalised with COVID-19, explained that her grandson had visited her at home. He did not want to leave because she seemed so unwell, and though his grandmother assured him that everything was fine, his concern prompted him to remain. He soon called the ordinary emergency number, but without success. After conferring with his mother, he called the coronavirus hotline, and things began happening rapidly. His grandmother was hospitalised due to his assessments and actions.

Several informants told similar stories about others initiating action on their behalf. Family members, friends, colleagues and even tenants became involved; several were driving forces regarding contacting healthcare services and advocating for tests and hospital admission. As this informant described:

*And my partner, who works in the healthcare services, told me ‘No! It can’t go on like this’. And she dragged me into a taxi in the middle of the night to the emergency room. They took a corona test. It was positive the day after, and I was admitted to hospital*. (Informant 17)

One informant, whose spouse was also ill, emphasised that their sons were vital to their survival. Without their sons’ close follow-up and prompt reactions to the worsening of their symptoms, they would not have been hospitalised in time. Another informant, who worked at a health centre, highlighted how her colleague’s response during a phone-call was crucial:

*The last time I called my doctor [to get a sick note], I was answered by the assistant or medical secretary. She asked me, ‘Are you breathing heavily?’ I said, ‘Yes, I am’. Then she said, ‘Well, you need to be hospitalized’. She called the hospital, which contacted me, and I was collected after that. And it really was a great relief to be admitted to hospital, because I started to wonder if I simply was about to die*. (Informant 6)

Our informants also described other, more unorthodox, actions taken by both informants and next-of-kin to access the health system. One informant’s son ordered an ambulance directly, without first consulting him or the emergency room. Another informant described how the health system denied her access to a test, despite the fact that she had a very high fever, of some duration. Her daughter-in-law called the emergency number to request an ambulance, which was denied due to her lack of a COVID-19 test. As her symptoms exacerbated to a very serious level, her tenant brought her to the emergency room without calling in advance. From that moment onwards, everything happened rapidly. Several other participants also described the efforts of those in their network as being vital for their survival.

A third informant was denied a COVID-19 test through the coronavirus hotline because she had not been to or met with anyone who had been to Austria on a ski holiday. This informant was frequently telephoned by a concerned friend, who accidentally knew that the informant’s neighbour had tested positive for COVID-19 after a recent Austrian holiday. When the informant called the coronavirus hotline once again, this time arguing that her neighbour had COVID-19, she was granted permission to receive a test.

## Discussion

Our main finding shows that the informants experienced both vulnerability and arbitrariness in their search for professional help when suffering from severe symptoms of COVID-19 at home during the early phase of the pandemic. This finding is based on the informants’ experiences of having a severe disease, their challenges around access to testing and hospitalisation, and the importance of receiving help from their next-of-kin.

The most striking finding in our study was that many of the informants reported needing to fight for access to necessary healthcare: first, to be assessed as needing a test, and second, to be assessed as needing professional treatment through hospitalisation. While some informants had comparatively easy access to testing and/or hospitalisation, most were denied both – even when they clearly displayed symptoms of a severe illness.

Our informants were infected in the beginning of the early phase of the pandemic, and very few met the national testing criteria outlined above. However, a public commission investigating how Norway handled the outbreak of COVID-19 has revealed serious, erroneous conclusions made by the health policy authorities in advance of the outbreak.^6^ They did not expect the rapid development of SARS-CoV-2 in Norway, in particular related to the spread of infection among the population. The commission noted the health care system’s unpreparedness towards a possible pandemic, which manifested in inadequate testing capacity and a lack of medical personalised protection equipment due to small contingency stocks.^6^ Such lack of preparation may be assumed to propagate downwards in the health system. This, in addition to a very contagious disease and lack of treatment knowledge among clinicians early in the pandemic, might explain our informants’ varying experiences in their encounters with the health services. For instance, the above issues may have caused some health professionals to perform overly strict assessments of patients seeking COVID tests and/or treatment.

Moreover, during the first 3 weeks of the pandemic, one of the two hospitals in which our informants were hospitalised had an average of 7 days from the debut of symptoms to testing,^29^ compared to the national median average of 4 days during that same period.^5^ This increased wait time does not reflect the experiences of helplessness and despair described by our informants, which arguably may have been exacerbated these emotions, judging by the symptoms and experiences they described in the interviews. The fight for access among our informants may therefore be understood as caused by a healthcare system that was unprepared for a pandemic, whose staff struggled to interpret the testing criteria during the first phase of the pandemic. Our findings related to the informants’ arbitrary access to healthcare must thus be seen in light of the lack of testing and hospitalisation capacity and expectations of a rapid increase in severe cases.^6^

Concerning our finding that informants experienced professionals’ assessments differently regarding access to testing and hospitalisation, we may understand this as being due to the informants’ arbitrary access to healthcare. Such arbitrariness stands in marked contrast to Norway’s core principle of equal access to public healthcare when needed, and the ethical principle of fair access to health service. This is closely linked to the ethical principle of justice,^9^ which entails that all people should be treated equally.^28^ Norway implemented criteria for prioritisation in order to secure fair allocation of healthcare, and hence avoid arbitrary access to healthcare. However, previous studies have shown that guiding criteria for priority settings may not be helpful when resources are strained.^9, 29^ In addition, outcomes of criteria for priority settings seem to be scarce.^30, 31^ According to Bærøe,^31^ guiding criteria may be even more difficult to use within a complex setting – this latter may characterise the situation in Norway in the early phase of the pandemic. Instead of following the national criteria, new criteria were introduced consecutively, based on experience numbers and expected development^6^ – criteria which also seemed to be very strict due to the severity of the situation. As argued above, we believe that the arbitrary access to health service encountered by our informants was due to the chaotic situation caused by a lack of knowledge and preparedness. However, ethical concerns that arise in the course of meeting patients’ needs should not be ignored by guiding criteria, even in the face of an unfamiliar disease and chaos. When these criteria become too rigid, professional care assessment becomes essential, because that care must respond to the individuals’ experience – and not solely to established criteria. Although knowledge of COVID-19 in the early phase was limited, health professionals did have knowledge related to general symptom assessments. We argue that their symptom assessments failed with many of our informants, even though such assessments should constitute basic competence among health personnel.

In the first phase of the pandemic, the criterion of severity seemed to be the most challenging for our informants to meet and healthcare professionals to evaluate. This criterion entails that the most severe condition should be given priority due to the risk of death or severe functional impairment.^10^ Issues around evaluating this criterion in the context of the pandemic have also been debated elsewhere,^14^ highlighting a lack of agreement on how the criterion of severity should be defined, argued for and operationalised. Previous research has also shown that one may be willing to take a greater risk – and hence stretch the criterion of severity – when resources are strained.^9^

Even in the early phase of the pandemic, it was well-known that COVID-19 was dangerous for older adults.^2, 4^ When considering our study findings in light of informants’ varying reports of how the severity of their clinical symptoms was assessed, it might seem that their vulnerability to both serious illness and the risk of death was not assessed in the best professional way by frontline health professionals, in terms of testing and hospitalisation needs. These experiences challenge our expectations regarding the provision – and receipt – of necessary healthcare for all. However, the formal responsibility of not being able to deliver appropriate health care under such circumstances is also of high importance, although out of scope for this paper.

Further, the criterion of severity should also be seen in relation to the criterion of benefit of intervention.^28^ The ability to consider the benefit of intervention, however, depends on one’s knowledge and research on the intervention. This was complicated in the early phase of the pandemic by a general lack of knowledge regarding the range of COVID-19 symptoms and also of treatment. Our findings reflect this, as some participants’ symptoms deviated from the more well-known COVID-19 symptoms (e.g. high fever and coughing); considering the benefit of intervention may also be challenging in a group of older people displaying multi-morbidities and frailty.^9^ Every health professional is nevertheless required to assess each patient’s symptoms with professional judgement. However, some informants were denied a test and sent home, despite displaying severe symptoms – a high fever for two to 3 weeks indicates a severe condition and is life-threatening, regardless of one’s disease status. We therefore wonder about such assessments made by health professionals and how strictly the criteria for both testing and hospitalisation should be followed, in light of our findings regarding the clinical assessments of the informants’ severe symptoms. Among health professionals, particularly nurses, the application of competent appraisal and the exercise of discernment are expected professional skills. It is also a significant aspect of providing care and doing morally good, when observing an ill patient.^32^ This indeed is in line with the ethical standards for nurses, for example, ‘the nurse contributes actively to meet the special needs of vulnerable groups concerning health and care services’ derived from the Norwegian Nurses Association.^12^ However, sometimes regulations, like national criteria, may hinder professional consideration and discerning judgement and the intention of doing good as an ethical principle. This also seems to have been the situation in the early phase of the pandemic, where very strict criteria seemed to override professional and discerning judgements. The guiding principles and the rather strict interpretation of the criteria governing access to emergency services highlight an important ethical concern. In many cases, the testing criteria did not sufficiently respond to critical symptoms of possible COVID-19 infections. With reference to care ethics and discussions about prioritisations and rights in healthcare services,^33–35^ care professionals need to understand the COVID-19 criteria in light of essential care ethics arguments, to avoid neglecting patients’ testing needs. Care ethics provide critical resources on the principles and practices of fair and just allocation of healthcare.^33, 34^ If some vulnerable individuals do not meet the prioritisation criteria but still require care, care ethics should inform these criteria so those in need are not neglected. Moreover, in accordance with international and national ethical standards, nurses are obliged to advocate for equity and social justice among patients who are not being offered adequate healthcare despite displaying obvious and severe symptoms yet are in great need of receive care.^11, 12^ The ethical standards describe a moral and professional standard and a commitment to support the needs of seriously ill patients, which some of our informants did not experience.

The strict testing criteria drove some of our informants, who did not meet that criteria, to enact unorthodox in order to get tested. We wonder, therefore, whether the criteria were interpreted too strictly, or if the serious lack of laboratory equipment in the very early phase drove frontline health professionals to act too strictly towards people who did not meet the criteria (e.g. having been to certain countries or in close contact with someone who had). While the testing criteria were altered and became more inclusive the day after the lock-down was implemented in Norway,^5^ it was in the unpredictable early phase when our informants were infected and encountered overworked health personnel.

Several of our informants described how they experienced themselves to be weak, confused and uncapable of self-care due to COVID-19. Some were at home, not understanding the severity of their condition. This may have been caused by a lack of knowledge, but also confusion (e.g. delirium), which has been reported particularly in older patients (>75 years) in one of the hospitals in this study.^36^

A final important finding concerns the importance for our informants of having someone who could advocate on their behalf for professional help, due to the severity of their condition. In the aftermath of the initial chaotic phase of the pandemic, the authorities admitted that some people most likely died at home without being recognised as a COVID-19 case.^6^ Although most of our informants were aware that they had a severe illness, many described how dependent they were on next-of-kin in order to access necessary healthcare. In particular, those whose spouse had COVID-19 described their role as quite stressful, with regards to fighting the system in order to obtain necessary healthcare for their loved one. Interestingly, this finding has not yet been reported elsewhere, as far as we know. When seen in relation to the correspondingly low response from the emergency services, the burden on next-of-kin seems to have become unnecessarily heavy.

### Strengths and limitations of the study

The main strength of this study is that it is part of a larger multi-centre study representing a high proportion of older patients, following their hospitalisation due to COVID-19.^4^ Another strength is the variation among the participants regarding both age and sex. The sample also produced a breadth and depth of data and confirmations of key statements, which informed our findings. In addition, six experienced researchers conducted all interviews in pairs, each of which also conducted initial analyses of the data and produced a consensus of the main themes. In this paper, four of these researchers worked in pairs while performing data analysis through a reflexive process to generate the final themes.

This study also has limitations. Our informants represent only two of the hospitals in Norway’s capital area producing a smaller convenience sample. The findings should therefore be interpreted with caution. Another limitation is that we conducted the interviews 6 months after hospital discharge, and the participants had to recall their experiences from when they were seriously ill. In addition, three of the interviews consisted of couples, who might have influenced each other’s statements; this also may have hindered certain thoughts from being voiced.

## Conclusion

The variety in participants’ experiences reveal an arbitrariness in healthcare access, despite Norway’s ethical principle of equal access to necessary healthcare. The priority criterion of severity seemed to be the most challenging in assessing patients. Even when dealing with an unfamiliar disease, health professionals’ assessments of symptom severity must be performed in accordance with their ethical obligation to applicate competent appraisal and the exercise of discernment; this is in line with care ethics and ethical standards for nurses. When they fail to do so, too much responsibility may be imposed on next-of-kin.
